# Communication in health and inter-professional collaboration in the care for children with chronic conditions*

**DOI:** 10.1590/1518-8345.4044.3390

**Published:** 2021-07-02

**Authors:** Maria Denise Schimith, Marta Regina Cezar-Vaz, Daiani Modernel Xavier, Letícia Silveira Cardoso

**Affiliations:** 1 Universidade Federal de Santa Maria, Departamento de Enfermagem, Santa Maria, RS, Brazil.; 2 Universidade Federal do Rio Grande, Escola de Enfermagem, Rio Grande, RS, Brazil.; 3 Universidade Federal do Pampa, Curso de Enfermagem, Uruguaiana, RS, Brazil.

**Keywords:** Communication, Child Health, Interprofessional Relations, Primary Health Care, Chronic Disease, Primary Nursing, Comunicación, Salud del Niño, Relaciones Interprofesionales, Atención Primaria de Salud, Enfermedad Crónica, Enfermería Primaria, Comunicação, Saúde da Criança, Relações Interprofissionais, Atenção Primária à Saúde, Múltiplas Afecções Crônicas, Enfermagem Primária

## Abstract

**Objective:**

to understand how health communication in the care of children with chronic conditions interferes with inter-professional collaboration.

**Method:**

a multicentric qualitative research. Data collection, carried out through interviews and observation, occurred from October 2017 to February 2018. For data organization, the NVivo software, version 12, was used. These data were analyzed from a dialectical perspective.

**Results:**

a total of 79 professionals were interviewed, including physicians and nurses in the Family Health Strategy. Essential markers for inter-professionality stand out, such as multi-institutional communication; the historical and political context of the municipalities; the bond between staff and families with children with chronic conditions; and active and purposeful communication.

**Conclusion:**

inter-professional collaboration is strengthened when the therapeutic plan of the child with a chronic condition is coordinated by the Family Health Strategy, plus the intention of communicating with the secondary sector. It is considered that the research included important issues, contributing to planning the work process in the Family Health Strategy.

## Abstract (es)

**Objetivo:**

comprender cómo la comunicación sanitaria en el cuidado de niños con afecciones crónicas interfiere en la colaboración interprofesional.

**Método:**

investigación multicéntrica, cualitativa. La recopilación de datos, realizada a través de entrevistas y observaciones, se llevó a cabo entre octubre de 2017 y febrero de 2018. En la organización de los datos, se utilizó el software NVivo versión 12. Los datos fueron analizados desde una perspectiva dialéctica.

**Resultados:**

fueron entrevistados 79 profesionales, incluidos médicos y enfermeros del programa Estrategia de Salud de la Familia. Se destacan marcadores esenciales para la interprofesionalidad, como la comunicación multiinstitucional; el contexto histórico y político de los municipios; el vínculo entre el equipo de profesionales y las familias con niños con afecciones crónicas y la comunicación activa y resolutiva.

**Conclusión:**

la colaboración interprofesional se fortalece cuando se coordina el plan terapéutico para niños con enfermedades crónicas a través del programa Estrategia de Salud de la Familia, y se verifica la intención de establecer comunicación con el sector secundario. Se considera que la investigación integró temas importantes y contribuyó a la planificación del proceso de trabajo en el programa Estrategia de Salud de la Familia.

## Abstract (pt)

**Objetivo:**

compreender como a comunicação em saúde na atenção de crianças com condições crônicas interfere na colaboração interprofissional.

**Método:**

pesquisa multicêntrica, qualitativa. A coleta de dados, realizada por meio de entrevista e observação, ocorreu de outubro de 2017 a fevereiro de 2018. Para a organização dos dados, utilizou-se o *software* NVivo versão 12. Esses dados foram analisados pela perspectiva dialética.

**Resultados:**

foram entrevistados 79 profissionais, entre médicos e enfermeiros na Estratégia Saúde da Família. Destacam-se marcadores essenciais para a interprofissionalidade, como a comunicação plurinstitucional; o contexto histórico e político dos municípios; o vínculo entre equipe e famílias com crianças com condições crônicas e a comunicação ativa e propositiva.

**Conclusão:**

a colaboração interprofissional é fortalecida quando o plano terapêutico da criança com condição crônica é coordenado pela Saúde da Família, acrescida da intencionalidade de comunicação com o setor secundário. Considera-se que a pesquisa integrou importantes temas, contribuindo para o planejamento do processo de trabalho na Estratégia Saúde da Família.

## Introduction

Primary Health Care (PHC) is responsible for longitudinal, integrated, and continuous monitoring of the population enrolled, and has a central role in coordinating care for patients with chronic conditions^1^. It involves other services so that comprehensive care can take place^2^. In Brazil, the model that guides its organization is based on the Family Health Strategy. In the Family Health Units (FHUs), communication in interpersonal relationships should constitute a different process, as it includes inter-professional relationships in these different areas of health, with inter-sectoral articulation, to effect the coordination of care^3^.

The collaborative practice in health care can be defined as the provision of services based on comprehensiveness, developed by health professionals from different areas. It aims to achieve the highest quality of care in the service network, involving patients, families, caregivers, and communities. Such practice can be included in clinical and non-clinical work, such as diagnosis, treatment, surveillance or communication in health, administration, and sanitary engineering. Inter-professional collaboration, on the other hand, is characterized by the sharing of health information, that is, communication between professionals in order to favor the care of the singularities for those who are inserted in a service in search for health care^4^.

In this research, chronicity in the child was considered as chronic diseases or conditions, with or without medical diagnosis, which need continuous monitoring by a health professional. It differs from the concept of Children with Special Health Needs (*Crianças com Necessidades Especiais de Saúde*, CRIANES) who are children who may or may not develop chronic conditions and have physical, developmental, behavioral or emotional needs and who need health services, in addition to those required by children in general^5^. It is noteworthy that CRIANES is not synonymous with a child with chronic conditions; however, in this article, the terms will be considered as complementary definitions.

It is understood that the increase in the complexity of health needs requires professionals to be prepared to work inter-professionally^6^. A research study revealed that CRIANES are more likely to require specialized care and have this demand not met, compared to children without special needs^7^.

The existence of teams committed to health care expands the possibilities for carrying out inter-professional actions^8^. However, a research study conducted with CRIANES revealed the weakness in home care by PHC, signaling the urgency of timely care for these children, so that there are links between the family and the health team^9^. The health workforce, based on inter-professional collaboration, is an essential mechanism to shape the effectiveness of the practice. For such, it is necessary to have an approach based on the population or on the determination of their needs, generating new inter-professional concepts and considering the way in which the health actions will be delivered to the population^10^.

For the collaborative practice to take place, the social and professional skills of the care managers were identified as fundamental, and communication, as a process of sharing information, is one of these skills^11^. The health work process, as a daily exercise, requires interaction and communication between professionals, and of these with the patients, family members or caregivers, as well as considering, in addition to the means-end type technical framework, the communicative competence, which concerns the social interaction^8^. 

However, there is still inadequate communication between professionals and users in the care of chronic conditions in children in PHC^12^. Inter-professional PHC teams have opportunities to improve collaboration, regardless of the organizational or political context in which they operate. For this, it is necessary that the team has a shared vision and objectives, formal quality processes, and information systems. In addition, the professionals need to feel part of the team^13^.

Extensive knowledge is available on the theme of “children with chronic conditions”; however, little is known about collaborative practices in the work process in the Family Health Strategy (FHS) with this population. From the research question of “how health communication in the care of children with chronic conditions interferes in the inter-professional collaboration?”, the aim is to understand how health communication in the care of children with chronic conditions interferes with inter-professional collaboration.

## Method

This is a qualitative and multicentric research study, developed by research groups from two federal universities. It has a descriptive-analytical character and is cross-sectional to the work process of FHU health professionals in two municipalities in the state of Rio Grande do Sul, Brazil.

The municipalities will be identified as Municipality A (MA) and Municipality B (MB). According to an estimate by the Brazilian Institute of Geography and Statistics (Instituto Brasileiro de Geografia e Estatística, IBGE), for 2019, MA had 282,123 inhabitants while in MB there were 211,005, and their demographic densities were 145.98 inhabitants/km^2^ and 72.79 inhabitants/km^2^, respectively^14^. They were chosen for facilitating access of the researchers. Collection took place from October 2017 to February 2018, consisting of a semi-structured interview and of observation.

The interviews were conducted at the FHU, recorded in mp3 audio, and transcribed. They presented questions about the professional trajectory; the actions developed by the professional, the team and inter-professionally; about how, with whom, and what they communicate, and about the purpose and difficulties encountered in communicating with family members and children with chronic conditions. All the physicians and nurses who were working were invited to participate. In MA, seven professionals did not accept to participate, while in MB twelve did not accept, two were not in the unit at the scheduled time, and it was not possible to contact eight individuals. At the end of each interview, the participants were asked how they would like to receive the research results, and it was clarified that the transcripts of their answers would be made available for checking.

The observations made after the interviews took place in different shifts, following the work process of nurses and physicians. In the field diary, scenes that were presented in the different modalities of actions developed by three teams in each municipality during the work in progress were recorded in a dense and meticulous manner. They were also recorded on an mp3 audio device. The audios of the observations were not transcribed in full, only used as a complement for the field diary, when necessary. 

Data was organized with the aid of the NVivo software, version 12, with no financial participation. The empirical data of the two collection techniques were articulated in the coding of the results, which were grouped by similarity or divergence, considering the different realities of the cities studied.

Subsequently, the empirical material was analyzed with a dialectical approach, inspired by the interpretative proposal^15^. A synthesis of the results was elaborated, listing argumentative cores, considered narratives. These were analyzed by two researchers. It was sought to reveal the history and the contradictions, in each studied reality and among the municipalities, in a contextualized manner, always considering their structure and organization. The narratives were sent by e-mail to the participants, so that they could be assessed and the disagreements be identified. This procedure guaranteed the credibility and reliability of the research. 

The ethical aspects of research with human beings were respected, according to Resolutions 466/2012 and 510/2016. The research was submitted to the Research Ethics Committee in the Health Area of a Federal University, being approved with Opinion NO. 65/2017, on October 20th, 2017, CAAE No. 4677317.0.1001.5324. Before starting data collection, the Free and Informed Consent Form (FICF) was analyzed and signed by the participants. In order to ensure the anonymity of the participants, the statements were coded with the letter E for interview (“Entrevista” in Portuguese), followed by the letter E for nurse (“Enfermeiro” in Portuguese) or M for physician (“Médico” in Portuguese), with the number of the interview sequence, plus the identification of the municipality (MA or MB). The excerpts from the field diary were identified with the day of the observation, followed by the identification of the municipality (MA or MB).

## Results

MA had 19 Family Health teams and 30 professionals, 15 physicians and 15 nurses, were interviewed. MB had 36 teams, with 50 professionals being interviewed. Among these, there were 30 nurses and 19 physicians, totaling 79 interviews, which lasted from 25 to 70 minutes, with 97 hours of observation. It is highlighted that not all the teams were complete; some had medical interns, with the preceptorship of another professional. 

The results revealed how health communication in the care of children with chronic conditions interferes with inter-professional collaboration. At the end, the word cloud was created, which supports the results found.

### Communication as an instrument of inter-professionality

The professionals from both municipalities noted the importance of care for children with chronic conditions to be carried out by the whole team, in a collaborative manner. Among them, there are differences regarding the scope of inter-professionality. *[…] Sometimes I need to make an appointment with the doctor, the community health agents bring us a lot of demand. In terms of visits, there’s no way out without them.* (EE1MA)*; […] We, for those special children, schedule the home visits, [...] to work with the family, with the management of the child, for us to look at how the family is doing, to detect any warning signs. There is the PSE program (Health at the School Program) [...] always the nurse, the dentist, they do actions there, but we also interact with the school. At the school of [rural town name] they have a school therapist, we work closely together. She detects children with delayed school development, which we evaluate if they need psychological assistance. So, we do this shared work.* (EM13MA)

In MA, communication involves inter-professionality between the team and the family. The school is also part of the actions they carry out. In MB, most of the professionals say that the team develops the role of care coordinator for children with chronic conditions, accompanied by the secondary sector and supported by a broad team.

*[...] The role of care coordinator for this family passes through us a lot. Our protagonism will cause us to make a comparison: if this child was in a sector of the city that doesn’t have a Family Health Unit, even if he/she has access to the secondary sector, the failure rate for this care will be much higher because there is just the secondary sector. Primary care [no FHS] cannot manage to support adequately. In the case of the Family Health Strategy, the success rate has to be better because you have a nutritionist, physical educator, physiotherapist, social worker, psychologist, doctor, nurse, and dentist. And access is not through a form, nor by password, it is due to the need of the person [inter-professionality]. She comes to the unit, seeks care and we join professional efforts to take care of this case. So, the matrix-related strategy is having five, six professionals discussing a therapeutic plan for this family. So, this will cause the person to really have full care.* (EM9MB)

In both municipalities, it was identified that the organizational model has implications in the management of the cases. While in MA the team has the CHAs, family, and school; MB has a Family Health Support Center (FHSC) structure, which expands inter-professionality. The MB teams manage to articulate inter-professionality with the FHSC, because in this municipality there are six FHSC teams, which serve the 36 FHS teams.

*[…] For these obese children, who are usually older, we generally call the FHSC to see, also, the part of the psychologist, nutritionist. It’s not something that is just between us, because, in addition to being less demand, they are more complicated cases. Because, for being a child, nutritionists, physical educators and psychologists usually take part.* (EM7MB)

In MA, some teams have the support of the Multi-professional Internship to expand care for children with chronic conditions. The testimonials reveal how inter-professionality happens and the benefits that arise from it.

*[…] We had a mother, for example, who didn’t do prenatal care in this FHU, she started bringing the child to weigh and she didn’t want anyone to come close, and then we found out that the child had Down [...] [diagnosed with Down syndrome] [...] in the weighing process I talked to her, the health agent talked, and everyone said the same, “but bring the child, but come, it’s important, let’s participate”, she started to come and started to bring the child to make follow-up with us. She was referred to a neurologist and was referred to pediatrics from there, for evaluation. [...] She went to the speech therapy too, because I have a speech therapist at the internship, so she evaluated and referred too. Precisely for that other look [from another profession] that we don’t have. If the speech therapist was not with me when the child yawned, I wouldn’t see that she had a heart-shaped tongue. So, these are things that the speech therapy, at the time “look over there, a heart-shaped tongue”.* (EE1MA)

The nurse highlights the importance of inter-professional collaboration allowed by the presence of the Multi-professional Internship. However, it is clear that it is still necessary to stimulate in the mother the bond with the FHU team.

### The interdependence between inter-professionality and bond

The difficulty of linking children with chronic conditions and their families with the FHU was also reported.

*[…] one difficulty we have is the fact that when people go to a specialized service, they think that they don’t need to follow-up the Strategy, […] so we started to get a follow-up, and these children, especially with more serious pathologies, the mothers say: “no, you don’t have to worry that he’s followed-up at the University Hospital”. [...] And then we explain that we won’t replace the specialized care, but we need to know, or you are at home and feel sick, I need to know what drug you use, then the people start to come, but it’s an ant job.* (EE1MA)

On the other hand, in MB, it was noted that the bond already exists, that the families of children with chronic conditions seek the FHU.

*We perform childcare, home visit, request material. For example, I have a girl, [name], every month I request the bottles for feeding, the probe, when she needs change. The mother had pre-eclampsia and ended up having a delivery at 26 weeks. The girl has several sequelae. And there is [name] with one year old, he had a stroke, he uses a wheelchair. So, we do all this follow-up part, their physiotherapy, referral. We also refer to the benefits that we have. Children end up receiving LOAS [Lei Orgânica da Assistência Social - Organic Law of Social Assistance]. So, we take care of the vaccines. We try to do a complete job for them. And we, too, have access, the parents, when they need, they come to the unit, ask for our work. They have a childcare agenda, too. The child always comes and leaves with the next scheduled appointment, it’s a continuous, programmed care.* (EE11MB)

While in MA it is difficult to link the family to the FHU, in MB, the link exists by the resoluteness felt by the family, as evidenced by the report of the work process. To explain the disparate realities, the historical and political context of the two municipalities with regard to the FHS coverage and the support of the FHSC teams is considered crucial.

### Written or telephone communication as an instrument for inter-professional collaboration

There are cases of chronic childhood conditions in which the nurse identifies the need for communication with other professionals or other services.

*[...] if I am doing the nursing consultation, I identified that this chronic patient is having some complication or is at risk, I will already call the doctor or will already call the dentist [...] we have already triggered the epidemiological surveillance.* (EE15MB)

This communication with other sectors differs in the reality of the researched municipalities. In MB, the professionals make explicit the need for communication and sharing the care with this sector.

*[...] we need to have contact with the reference sites, with other professionals, the service is not restricted only to here, with the professionals of the unit. In reality, we work with other sectors. Arrives here, I make contact with [name of the university], “Oh, I’m in a situation like this”. Sometimes the child arrives in a more precarious situation, we already make other referrals. We have the FHSC, the social worker is already mobilized, so it depends on each context, each situation.* (EE16MB); *[…] And the care of children with chronic illness requires more joint care with the secondary sector.* (EM9MB); *[…] With the team, with other sectors, with other professionals. Sometimes, like this, if the team itself has doubts, like that, they call a specialist to seek some guidance. And they make the necessary referral. In fact, they exchange some ideas.* (EE16MB)

The MB nurse demonstrates how the case is matrixed. For this, written communication brings PHC closer to the secondary sector, favoring comprehensive care.

*[…] They have the consultation with the doctor, but they’re also children who are in the scheme, like this, or they already consult with the pneumopediatrician. They manage to consult with their pediatrician. And then more come or to renew [prescription] or in crisis. There are children with autism, I have them here, but then there is also the [name of autism association] and there is Caps I [child]. Caps I answers. The [name of autism association] provides care. And then the doctor over there sends them saying “oh that patient”, gives the history and asks the doctors to renew the prescription. And the doctors, whenever there is any complication, assist them, normally. And they always have free access, no need for scheduling.* (EE1MB)

In MA, however, there are weaknesses in counter-referrals. The lack of communication with the secondary sector, identified as lack of return, was well evidenced in the testimonies of the professionals from that municipality.

*[…] I always try to have a counter-referral from a specialist, that I, in three and a half years, has never been sent me a counter-referral, of all the referrals that I write “we request, please, a counter-referral”, to see their response, and we don’t have any, it does not exist.* (EM5MA) *[…] there are children followed-up in the HU, too, but we don’t receive feedback. Sometimes it’s the patient who brings it. And the patient brings the little paper, but we don’t have any feedback from the institution, and that is necessary, because sometimes we don’t know what was defined.* (EE10MA)

When mentioning that the “patient brings the little paper”, the nurse refers to the patient care record at the University Hospital. In this case, the return of the case occurs informally, depending on the responsibility of the patient/family.

In MA, some professionals claim that the child with chronic condition belongs to the specialized sector, without revealing the need for communication among the sectors.

*[child with neurological] problem we have, the same thing is done, home visit (by the CHA). Researcher: -Do they have link with other services? Doctor: -Yes, they usually have a link there with the university hospital, with the pediatric neurology of the university hospital. Researcher: -Don’t they stay here with you? Doctor -No, no.* (EM11MA)

Inter-sectoral communication, being written or by phone, occurs differently in the two municipalities, interfering in inter-professional collaboration. The data confirm the influence of the municipal context, in this case, involving the FHUs and the specialized services.

### Null or contradictory verbal communication

Observation data reveal the difficulty for communication in complex cases. In such situations, it becomes null.

*[...] In the last child to be seen in the childcare group, the nurse asks the mother: - got better? The mother answers that the husband drinks [alcoholic beverage] everyday, saying he’s “commemorating” the birth of his son. She laughs. The nurse asks: - every day? The mother says her husband claims he’s young, that drinking is okay, that he’ll stop whenever he wants. The nurse doesn’t provide any guidance. After the mother and child leave the space, the nurse explains that the case is delicate, that her family started to be assisted recently, as her husband is an alcoholic and violent, and that, little by little, the children began consultations in the Unit. The nurse explains that it’s very difficult to work with families that have these problems [alcoholism], that this has to be an ant job.* (Field diary, 01/18/2018, MB)

It was verified that the MB nurse did not provide guidance to the mother at the time observed, but revealed knowing the case. In addition, she made it clear that the teams need to act slowly and continuously to link cases of child vulnerability.

The contradictory communication between the team in the health care of children with chronic conditions was observed in MA, revealing the difficulty of the professionals to develop inter-professionality. Childcare developed by a nurse and Community Health Agent. *Overweight boy, three years old, 27 kg, 106 cm tall. The mother requests medication to stop breastfeeding, the nurse and the CHA ask the frequency of breastfeeding, the mother answers that it’s only at night. At the same time, the nurse and the CHA say that it’s not necessary to take medicine, just stop offering. The mother informs that during the day he takes a bottle, but at night the mother-in-law tells her to give the “teat” because the boy cries and the grandmother is tired because she works in construction. The nurse advises to offer milk to the child in the cup, the CHA disagrees with the nurse, and says: - “he’s a baby, he can take the bottle, yes” and even tells the nurse: - “for your children I guarantee that you give a bottle!” The nurse starts to guide brushing for the child.* (Field diary, 11/20/2017, MA)

It is revealed that contradictory communication between two professionals prevents inter-professionality from happening, especially in the presence of the patient. The importance of active and respectful communication between the team is highlighted, as well as between the team and the users. This communication should permanently be a theme of education, with a view to inter-professional collaboration.

**Figure 1:**
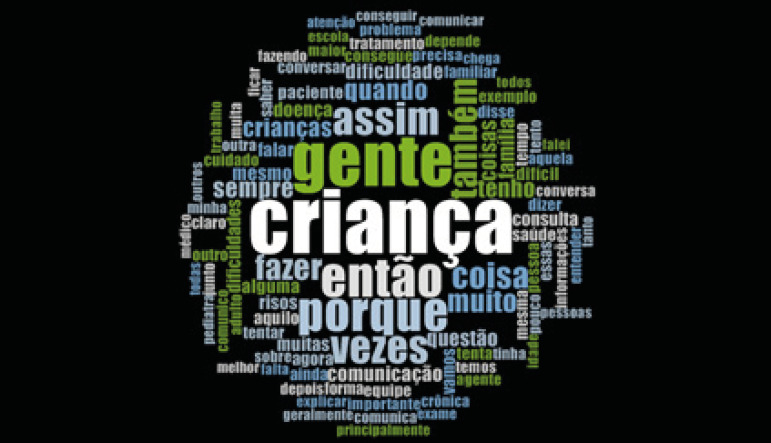
Word cloud generated from the NVivo software, version 12 - Frequency query for words present in the interviews and in the field diaries.

The results herein presented are supported by the word cloud (Figure 1), which contains the repeated words from the collected data set. The centrality of the word *criança* (child), surrounded by the words *equipe* (team), *comunicação* (communication), *informações* (information), *família* (family), *cuidado* (care), *conversa* (conversation), *trabalho* (work), *tratamento* (treatment), and also *dificuldades* (difficulties), validate the analysis of the submitted empirical material.

## Discussion

The research participants indicated that communication, in the team as well as with other institutions, is necessary to develop inter-professional collaboration with the child with a chronic condition, due to the fact that they are, in general, complex cases, requiring diverse professional knowledge. 

The MB team professionals cited the FHSC as a possibility to expand inter-professionality. In MA, the teams have the family, the school, and the Multi-professional Internship. It is noticed that the implementation of the FHSC teams can help in the inter-professional practice in the FHS, although the way of organization and the proactivity of the professionals, equally, produce an impact related to this.

A study identified that there are gaps in the health education of professionals to work in PHC in accordance with the principles of the Unified Health System, favoring comprehensive care for the user^16^. It was verified that there are possibilities for collaborative integration in the FHS, but it is necessary that the institutions, through their managers, approach and recognize their spaces and their functions. The professionals are responsible for the effort and commitment in the search for the inter-professionality. 

Inter-professionality with the secondary sector was identified in the statements of the MB professionals, since they strive to maintain communication with specialized sectors, and sharing the care for children with chronic condition seems to be occurring from a major integration. While MA awaits the counter-referral form, MB calls and exchanges ideas with the specialists.

The intentionality of the communication with the secondary sector and the practice of self-calling the coordination of the therapeutic plan, considering that the child with a chronic condition is under the responsibility of the FHU, allows us to think that integrality in the care of children with this type of problem can be achieved in the municipalities, as evidenced in MB. It can be said that, in MA, this intentionality is not shared between PHC and the secondary sector

In a research study conducted with caregivers of children with type 1 diabetes mellitus, the specialized service was identified as a regular source for care and a place for best health practices. According to the study, the greater contact of the participants with the specialized service, as well as their readiness, can be related to the choice for this regular source of care, revealing the weaknesses of PHC actions and services^17^. About CRIANES with type 1 diabetes mellitus, a research study revealed that the families spend a lot of time daily in the care of their children, compared to CRIANES without a diagnosis^18^. 

A review also confirms that parents of children with chronic conditions have significant and distinct needs for support throughout the care of their children^19^. Still, parents of CRIANES usually seek information from other parents in order to face the challenges in terms of caring for their children. They also need emotional support, and use social media for this^20^. Multidisciplinary teams have space to ensure that the interventions are viable, relevant, and accessible to the parents of these children^19^.

In MA, an absence of counter-referral was identified, making communication with the secondary sector impossible. However, PHC is responsible for integrating the care received by the child from other points of care. For this, communication and the proper functioning of the assistance flow are essential for the services to support the families during the children’s illnesses^21^. A research study carried out at PHC in Belo Horizonte, Minas Gerais, Brazil, corroborates so, stating that the work developed in a fragmented way in the team, the inefficiency of referrals and counter-referrals, as well as the difficulty of working with other health sectors, can impair continuity of care for children with chronic conditions^22^. 

Likewise, the specialized service has been identified as a regular source of health care in another research study^23^. The health needs of children with chronic conditions are multifaceted and require various disciplines and services, developed in an inter-professional manner^2^^,^ 24. In this way, integration requires from the services to fortify the communication capacity among them, the definition and the attribution of the actions for each service. Moreover, it requires managers to commit to the policy and legislation, institutionalizing integration actions of the services and of the longitudinal care actions^24^.

It is necessary to recognize that inter-professional collaboration is essential for implementing a pediatric primary health care model intending to attain, as a result, the health and well-being of the population^25^. For this purpose, it is indispensable to face the absence of counter-referrals identified by the MA professionals, seeking other ways of communication, which respond to the needs identified by the teams for the construction of the therapeutic plan for children with chronic conditions. 

A study highlighted that, after sharing goals between two sectors, community pharmacy and family medicine team, in the United States, the teams jointly developed communication strategies to manage the care for patients with hypertension and diabetes mellitus. The greatest result of the project was considered to be the formation of a collaborative team between these sectors, which keeps working together in other initiatives, in which the patient is the center of the care provided^26^. Another research study identified that being clear about the roles of each professional, as well as sharing cases, using communication skills, having confidence, and receiving support, are factors influencing the interaction among nurses and physicians from diverse sectors^27^.

Inter-professionality requires the professionals to communicate effectively and respectfully. Effective communication and understanding of the professional responsibilities are core competencies of a patient-centered collaborative practice^28^. In this context, the health professionals who wish to act as inter-professional teams must invest considerable time, in order to seek to understand the purpose and direction. Yet, they need to be clear about the tasks in which they work^29^.

The research did not interview users, being identified as a limiting factor. Future works are suggested that aim to understand the perception of the family members of children with chronic conditions about inter-professional communication between the FHS and the different services they need.

It is of special note that the present research study contributed to the advancement of scientific knowledge regarding the theme of children with chronic conditions in PHC, since it highlighted the precious elements of inter-professional collaboration. It was found that communication between the health sectors depends on the active movement of the professionals, revealing that it is possible for the FHU team to assume responsibility for coordinating the therapeutic plan.

## Conclusion

This research allowed us to understand how health communication in the care of children with chronic conditions interferes in inter-professional collaboration. It is concluded that the essential markers for inter-professionality are the following: the amplitude of inter-professionality, given by plurinstitutional communication; the historical and political context of the municipalities; the bond between staff and families with children with chronic conditions; and active and purposeful communication. It is essential to focus on such factors to favor inter-professional collaboration.

The coordination of the therapeutic plan and the responsibility of the FHU for children with chronic condition, with inter-professional collaboration, plus the intention of communicating with the secondary sector, were the most important evidences of this research. These are factors that differentiate services. In this context, communication is essential in order for inter-professionality to operate in the care of children with chronic conditions, favoring plurinstitutionality and inter-sectoriality.

Communication is a skill that needs to be learned and valued among the health care professionals. It is the task of the managers and professionals to think about strategies that facilitate and establish the care-related flow among the various services. 
